# CRISPR/Cas9-Mediated Targeted Mutagenesis of *CYP93E2* Modulates the Triterpene Saponin Biosynthesis in *Medicago truncatula*

**DOI:** 10.3389/fpls.2021.690231

**Published:** 2021-07-26

**Authors:** Massimo Confalonieri, Maria Carelli, Silvia Gianoglio, Andrea Moglia, Elisa Biazzi, Aldo Tava

**Affiliations:** ^1^Council for Agricultural Research and Economics, Research Centre for Animal Production and Aquaculture, Lodi, Italy; ^2^Department of Agricultural, Forest and Food Sciences, Plant Genetics and Breeding, University of Torino, Grugliasco, Italy; ^3^Instituto de Biología Molecular y Celular de Plantas, Consejo Superior de Investigaciones Científicas, Universidad Politécnica de Valencia, Valencia, Spain

**Keywords:** *Medicago truncatula*, triterpene saponin, CRISPR/Cas9, secondary metabolism, genome editing, cytochrome P450

## Abstract

In the *Medicago* genus, triterpene saponins are a group of bioactive compounds extensively studied for their different biological and pharmaceutical properties. In this work, the CRISPR/Cas9-based approach with two single-site guide RNAs was used in *Medicago truncatula* (barrel medic) to knock-out the *CYP93E2* and *CYP72A61* genes, which are responsible for the biosynthesis of soyasapogenol B, the most abundant soyasapogenol in *Medicago* spp. No transgenic plants carrying mutations in the target *CYP72A61* gene were recovered while fifty-two putative *CYP93E2* mutant plant lines were obtained following *Agrobacterium tumefaciens*-mediated transformation. Among these, the fifty-one sequenced plant lines give an editing efficiency of 84%. Sequencing revealed that these lines had various mutation patterns at the target sites. Four T0 mutant plant lines were further selected and examined for their sapogenin content and plant growth performance under greenhouse conditions. The results showed that all tested *CYP93E2* knock-out mutants did not produce soyasapogenols in the leaves, stems and roots, and diverted the metabolic flux toward the production of valuable hemolytic sapogenins. No adverse influence was observed on the plant morphological features of *CYP93E2* mutants under greenhouse conditions. In addition, differential expression of saponin pathway genes was observed in *CYP93E2* mutants in comparison to the control. Our results provide new and interesting insights into the application of CRISPR/Cas9 for metabolic engineering of high-value compounds of plant origin and will be useful to investigate the physiological functions of saponins *in planta*.

## Introduction

**Figure 1:**
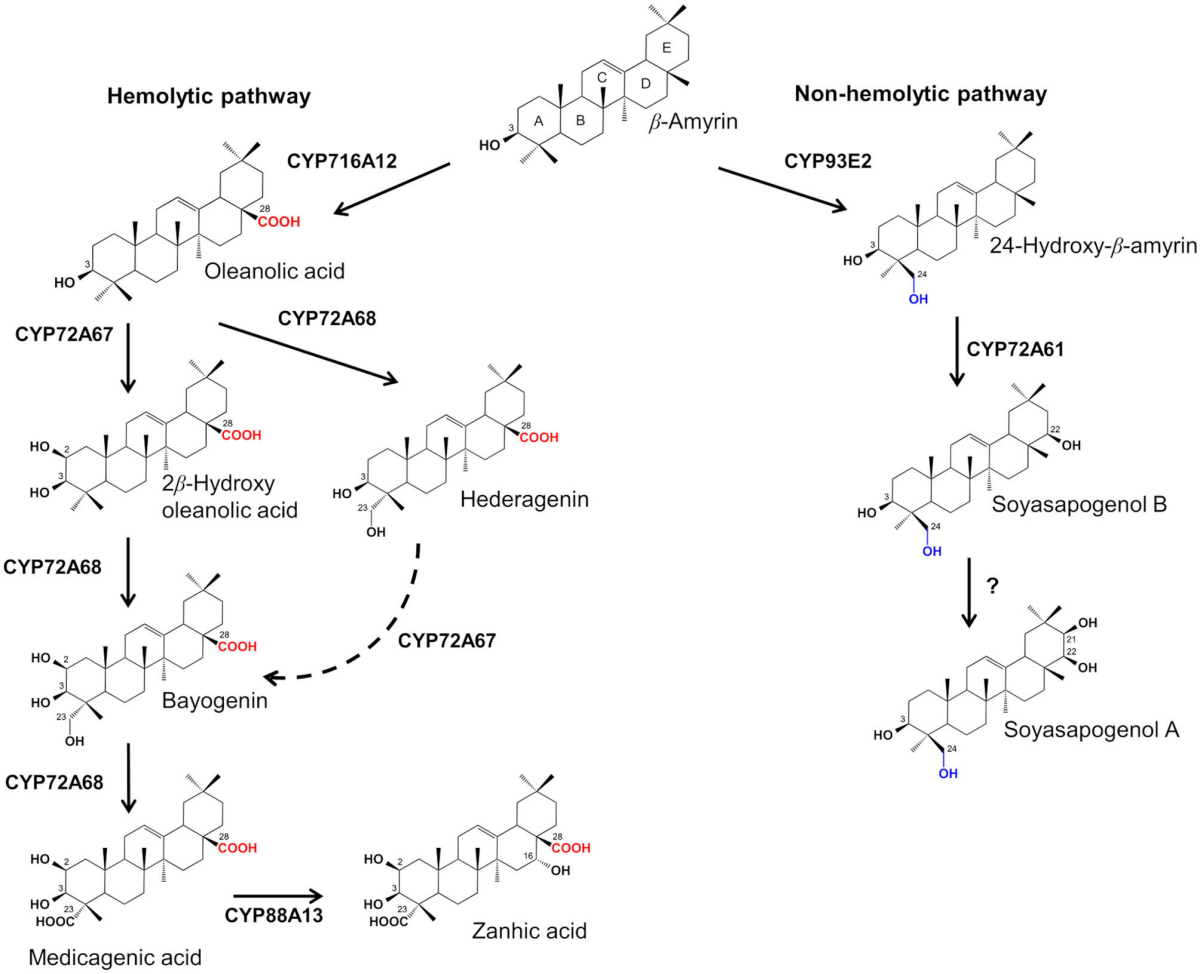
Biosynthetic pathway of triterpene sapogenins in *Medicago truncatula*.

Saponins are a large group of triterpene or steroid glycosides common to many plant species (Haralampidisis et al., 2002) exhibiting a wide spectrum of biological and pharmacological activities (Sparg et al., 2004; Tava and Avato, 2006; Augustin et al., 2011; Moses et al., 2014a). These properties have, in the last decades, stimulated research on triterpene saponin biosynthetic pathways using *Medicago truncatula* (barrel medic) as model plant species (Carelli et al., 2020). In the *Medicago* genus, saponins are a complex mixture of olean-type glycosides with medicagenic acid, zanhic acid, hederagenin, bayogenin and soyasapogenols A and B as aglycones (Tava and Avato, 2006). All the saponins from *Medicago* spp. possess the triterpenic pentacyclic nucleus belonging to the class of β-amyrin (Tava et al., 2011). Subsequent modifications in specific positions of the basic β-amyrin backbone by oxidative reactions mediated by different cytochrome P450 (CYP450s) oxygenases lead to distinct sapogenins (Tava et al., 2011) (Figure 1). The sapogenins are then subjected to glycosylation reactions mediated by glycosyltransferases to give the different saponins (Augustin et al., 2011). Based on the position and the oxidation degree of their functional groups, it is possible to distinguish two different classes of sapogenins in *Medicago* spp.: soyasapogenols are characterized by a hydroxyl group at the C-24 position and are defined non-hemolytic sapogenins while hemolytic sapogenins have a hydroxyl or carboxyl group at C-23 and a carboxyl group at C-28 (Tava et al., 2011) (Figure 1). The hemolytic and non-hemolytic sapogenins are synthesized through independent biosynthetic pathways, and have different chemical structures, biological activity and distribution in plant organs (Tava et al., 2011; Carelli et al., 2020). In particular, hemolytic sapogenins such as bayogenin, hederagenin, medicagenic acid, zanhic acid and their glycosides, which represent the most abundant saponins in the *Medicago* genus, showed significant biological and pharmacological properties, including fungistatic, insecticidal, antimicrobial, antifeeding, apoptotic, nematocidal, anthelmintic, cytotoxic, hypocholesterolemic, antiatherosclerotic, neuroprotective and anticarcinogenic activities (Avato et al., 2006, 2017; Tava and Avato, 2006; Argentieri et al., 2008; Balestrazzi et al., 2011; Rafińska et al., 2017; Maestrini et al., 2019, 2020; D'Addabbo et al., 2020). On the other hand, the soyasapogenol saponins are lacking of hemolytic characteristics and for this reason they have a low biological and pharmacological activity (Tava and Avato, 2006; Argentieri et al., 2008; Rafińska et al., 2017; Carelli et al., 2020). Due to their properties, hemolytic saponins from *Medicago* spp. are valuable specialized metabolites to be employed in the agro-industry and for pharmaceutical applications (Ellen et al., 2007; Bora and Sharma, 2011; Rafińska et al., 2017).

In *M. truncatula*, almost all of the P450s that are involved in the hemolytic sapogenin biosynthesis have been identified and their functional role has been characterized (Carelli et al., 2011; Fukushima et al., 2011, 2013; Biazzi et al., 2015; Ribeiro et al., 2020). However, the *in planta* function of a limited number of these P450s was determined. To date, two CYP450s key genes have been identified in the non-hemolytic sapogenin pathway in *M. truncatula*, and their role has been characterized only *in vitro* using a yeast heterologous expression system: CYP93E2 catalyzes the C-24 hydroxylation of β-amyrin (Fukushima et al., 2011), after which CYP72A61 catalyzes the hydroxylation of 24-hydroxy-β-amyrin at C-22-β position (Fukushima et al., 2013) resulting in the biosynthesis of soyasapogenol B, the most abundant soyasapogenol in the genus *Medicago*.

Mutants are pivotal for understanding gene function and to elucidate metabolic pathways and can be a valuable resource for crop improvement as well (Liu et al., 2018). Mutant legume plants with modified sapogenin composition have been described. By combining an activation tagging method and a reverse genetic TILLING approach, Carelli et al. (2011) identified *M. truncatula* plant lines mutated in *CYP716A12*, a key gene in hemolytic sapogenin biosynthesis. These mutants were unable to produce hemolytic sapogenins, synthesized only soyasaponins and showed severe phenotypic effects such as reduced plant growth and seed germination delay. In addition, another *M. truncatula* mutant line carrying a loss-of-function allele in *CYP72A67* was discovered in the same TILLING collection. *CYP72A67* mutant plants showed altered hemolytic sapogenin production without significant effects on plant growth and reproduction (Biazzi et al., 2015). Krishnamurthy et al. (2019), using a high-density soybean mutant library, isolated *GmBas1, CYP93E1*, and *CYP72A61* mutants. In particular, they showed that *CYP93E1* mutants were deficient in total saponins and accumulated a novel saponin having sophoradiol as aglycone. Suzuki et al. (2019) assessed in *Lotus japonicus* the *in planta* roles of two P450s (LjCYP93E1 and CYP716A51) in triterpenoid biosynthesis by performing gene loss-of-function studies, and showed that *LjCYP93E1* homozygous mutant plants did not accumulate soyasapogenols. However, at present no mutant plant lines lacking non-hemolytic sapogenins have been reported and characterized in *Medicago* spp. Moreover, their specific physiological role in plant growth and development is still unexplored.

Innovative genetic engineering methods have been recently developed to modify specific sites in targeted genes and generate knock-out mutants (Sedeek et al., 2019; Confalonieri and Sparvoli, 2020). Of these, the CRISPR (Clustered Regularly Interspaced Short Palindromic Repeats)/Cas9 system due to its high efficiency, simplicity and specificity has become a powerful and effective tool for targeted genome editing and research into gene function in plants (Doudna and Charpentier, 2014; Manghwar et al., 2019). This technology makes it possible to induce point mutations in one or some target sequences simultaneously, as well as to introduce new genetic variants by homology directed recombination (HDR) or to target and modify transcription. At present, few studies have been published related to the CRISPR/Cas9-mediated genome editing on *Medicago* spp. (Michno et al., 2015; Curtin et al., 2017, 2018; Meng et al., 2017; Gao et al., 2018; Wang et al., 2018; Yu et al., 2018; Wolabu et al., 2020), but none of them involved specific genes associated with the saponin metabolism.

In this study, we used the CRISPR/Cas9 technology in the model legume *Medicago truncatula* to knock-out *CYP93E2* and *CYP72A61*, the two key genes of the non-hemolytic sapogenin pathway, in order to elucidate their specific roles in saponin biosynthesis *in planta* and explore their possible involvement in plant growth and development. A number of new allelic *CYP93E2* variants were obtained while no *CYP72A61* mutants were produced. Selected *CYP93E2* mutant lines were analyzed for sapogenin content and plant growth performances. The present work illustrates the feasibility of using CRISPR/Cas9-targeted mutagenesis to knock-out *CYP93E2* in *M. truncatula*. We demonstrated for the first time the success of this strategy to block the metabolic flux to non-hemolytic saponins, highlighting the potential for converting β-amyrin to hemolytic saponins.

## Materials and Methods

### Plant Material and Growth Conditions

The *Medicago truncatula* cultivar Jemalong (M9-10a genotype) was used for tissue culture and transformation. This genotype was selected by Neves (2000) for its high regeneration potential via somatic embryogenesis. M9-10a plantlets were grown and micropropagated *in vitro* as described by Faè et al. (2014). *In vitro* cultures were kept in a growth chamber at 22–24°C with 16 h photoperiod of 65–75 μmol m^−2^ s^−1^ under cool white fluorescent lamp.

### SgRNA Design and CRISPR/Cas9 Vector Construction

gRNA sequences were selected using the CRISPR-P webtool (http://crispr.hzau.edu.cn/CRISPR2/), taking into account gRNA position and their on-target scores, using the *Medicago truncatula* Mt40v2 genome as reference. Putative off-target loci were further evaluated with Cas-OFFinder (http://www.rgenome.net/cas-offinder/) using the same reference genome, looking for up to five mismatches. Vector assembly was carried out according to the GoldenBraid cloning standard (https://gbcloning.upv.es/). The GB parts used in the assembly are listed in Supplementary Table 1. The final pCambia pDGB3 omega1 vectors, pDGB3 omega1 *nptII*-gRNA1-gRNA2-*Cas9* (*CYP93E2*) and pDGB3 omega1 *nptII*-gRNA1-gRNA2-*Cas9* (*CYP72A61*), were transformed in EHA105 *A. tumefaciens* electrocompetent cells. As a negative control pDGB3 omega1 *nptII*-*Cas9* vector was used.

### Plant Transformation and Generation of Transgenic Barrel Medic Lines

The *Agrobacterium tumefaciens*-mediated transformation procedure of M9-10a leaf explants was based on the protocol described by Confalonieri et al. (2009). Briefly, wounded leaflets were dipped into the EHA105 *Agrobacterium* suspension (OD_600_ = 1.6) and then co-cultivated for 5 days on solid embryo induction medium (EIM) in the dark. Subsequently, the inoculated explants were transferred on the same medium supplemented with carbenicillin (500 mg/L) and kanamycin (100 mg/L). After 3 weeks, leaf explants with embryogenic calli were transferred and subcultured on embryo proliferation medium (EPM) with the same antibiotic concentrations, until somatic embryos could be isolated. The kanamycin-resistant embryos were then grown on selective Embryo Conversion Medium (ECM) to develop into plantlets. Before acclimation and transplanting into pots to the greenhouse, putative mutant and control transgenic plants were subjected to antibiotic screening and surviving shoots were transferred to root-inducing medium for regeneration of complete plants.

### Screening for Mutations Induced by CRISPR/Cas9 in *CYP93E2* Gene

Genomic DNA was extracted from leaves of analyzed plants using a genomic DNA extraction kit (Sigma-Aldrich) following the manufacturer's protocols. The genomic DNA was used as template in a PCR amplification reaction using primers that were designed to amplify the CAS9 gene and 600 and 650 bp amplicons containing the specific sg1RNA and sg2RNA target sequences, respectively (Supplementary Table 2). PCR products were treated with Exonuclease I and FastAp thermo-sensitive alkaline phosphatase, purified by precipitation with ethanol:EDTA and sequenced using the Mycrosynth Barcodes Easy run service (Microsynth SeqLabDE - 37081 Göttingen). Sequences were analyzed using the TIDE web tool (https://tide.nki.nl/ Brinkman et al., 2014) in order to evaluate editing efficiency at the target sites as well as pattern of mutations.

### Greenhouse Evaluation of *CYP93E2* Mutant Lines

To evaluate the effects of CRISPR/Cas9 editing of *CYP93E2* on plant growth features and sapogenin content, the well-rooted *in vitro CYP93E2* mutant and control transgenic plants (T0 generation) were transplanted into pots (12 cm diameter) containing a mixture of peat and soil (1:2) and acclimatized for a week under growth chamber conditions (16 h of light at 24°C and 8 h of dark at 22°C), until shoot growth started. Subsequently, they were transferred in greenhouse at the beginning of the growing season at a temperature of 20–24°C, with a natural photoperiod. All the agronomic techniques were performed following the standard trial procedures. A total of 20 transgenic plants were analyzed, corresponding to 4 biological replicates of four independent *CYP93E2* mutants (T81 8, T83 1, T83 8, and T83 14) and a control transgenic line. The experimental design of the greenhouse experiment was a randomized complete block design with four replications of single-plant experimental units. Leaf, stem and root samples were collected from start flowering plants. The following parameters were evaluated: number of days from transplanting to flowering, fresh and dry weight of leaf, stem and root biomass. For dry weight measure, fresh samples were frozen at −80°C and then lyophilized.

### Analysis of the Sapogenin Content in the Plant Material

Leaves, stems and roots were separately harvested from each plant grown in a greenhouse and independently analyzed. Four replicated plants from all *CYP93E2* mutant lines and a control line were used for biochemical determination. Immediately after sampling, fresh plant tissues were frozen at −80°C and then lyophilized. All the aglycone moieties were considered as representative of the corresponding glycosides and evaluated by chemical methods. Sapogenins were obtained after acid hydrolyses of the corresponding saponins, as reported by Tava et al. (2020), identified by GC/MS and quantitated by GC/FID as their methyl/silyl derivatives considering all the obtained artifacts (Tava et al., 2017) (Supplementary Figure 1). Hundred milligram of plant material were treated with 50 ml of 2 M HCl in 50% aqueous methanol under reflux for 8 h. Uvaol (0.2 mg) was added as internal standard, methanol was removed *in vacuo* and the aglycones were extracted with ethyl acetate (3 × 20 ml). The organic solution was dried under anhydrous Na_2_SO_4_ and evaporated to dryness. Aglycones were dissolved in 0.5 mL of MeOH, treated with CH_2_N_2_ for 15 min and then the solvent eliminated under a stream of N_2_. Silylation was performed on the methylated sapogenins using 0.2 mL of a mixture of pyridine-hexamethyldisilazane-chlorotrimethylsilane (Merck) 2:1:1 at 70°C for 10 min. Samples were diluted with isooctane and used for GC/FID and GC/MS analyses. GC/FID analyses were performed with a 30 m × 0.32 mm, 0.25 μm i.d., DB-5 capillary column. Injector and detector temperatures were set at 350°C; the oven temperature program was: 90°C for 5 min, increased at 20°C/min to 250°C for 1 min and then increased at 4°C/min to 350°C for 15 min. Samples (1 μL) were injected in the “splitless” mode. He was the carrier gas with a head pressure of 12.2 psi. GC/MS analyses were carried out using a 30 m × 0.25 mm, 0.25 μm i.d., Elite-5MS capillary column using the same chromatographic conditions as for GC/FID. Mass spectra were acquired over 50–850 amu range at 1 scan/s with ionizing electron energy 70 eV. Transfer line 300°C, carrier gas He at 1.2 mL/min. Retention times and MS spectra were compared to those of previously identified sapogenins, considering all the formed artifact compounds as reported in Tava et al. (2017). All samples were analyzed in triplicate.

### Expression Analysis of Saponin Metabolic Pathway-Related Genes in *CYP93E2* Mutant Lines

Expression analysis of saponin pathway-related genes was performed by quantitative real-time PCR. *CYP93E2, CYP72A61, CYP716A12, CYP72A67, CYP72A68, CYP88A13*, and *BAS1* genes were analyzed. Total RNA was extracted from fully developed trifoliate leaves and roots of mutant and control plants harvested at the beginning of flowering, using the Nucleospin RNA plant kit (Macherey-Nagel, Düren, Germany) according to the manufacturer's protocol. For each transgenic line, four plants (biological replicates) were analyzed. cDNA was synthesized using the iScript cDNA Synthesis Kit (Bio-Rad) starting from 1 μg of RNA. cDNA was diluted 1:5 and 3 μl was used as a template in a 10 μl reaction containing 5 μl of SSoFast EvaGreen supermix (Bio-Rad) and specific primers for the selected genes (Supplementary Table 2). Thermal cycling conditions were: 3 min of initial denaturation at 95°C, 50 cycles of denaturation (95°C; 25 s), annealing and extension (59.5°C; 3 s) and a final melting analysis from 55 to 95°C with a 1°C increase at each step. The *Medicago sativa* translationally controlled tumor protein Msc27 (Pay et al., 1992) and Actin control genes were amplified in the same conditions. qRT-PCR reactions and analyses were carried out on three replicates each in a Rotor Gene 6000 (Qiagen) as previously described (Carelli et al., 2015). The gene expression levels were calculated by the comparative Ct method using the equation E = 2^−ΔΔCt^. ΔΔCt values were calculated as differences in ΔCt between edited lines (leaves or roots) and the roots of control line.

### Statistical Analysis

All data were subjected to the Analysis of Variance (ANOVA). Mean comparison between control and *CYP93E2* mutant lines was done by Dunnett's Test (*p* < 0.05) (SAS Proc GLM, SAS Institute Inc., Cary, NC).

## Results

### CRISPR/Cas9 Design to Knock-Out *CYP93E2* and *CYP72A61* Genes in Barrel Medic

For each of the target genes, two specific gRNAs were designed, directed at their coding sequences. For each gene, one gRNA was directed at the C-terminal Heme Cys domain, while the other was chosen to be as close as possible to the 5' end of the coding sequence, in order to ensure the early disruption of translation. The selected gRNAs also did not pose any significant risk of off-target activity, as inferred by the *in silico* prediction of putative off-target loci in the *M. truncatula* genome (Supplementary Data 1). Each gRNA was put under the control of the *Arabidopsis* U6-26 promoter. For each target gene, the two gRNAs were assembled in a pCambia vector together with the Cas9 endonuclease and the *nptII* selection marker for kanamycin resistance.

### Production of *CYP93E2* Mutant Barrel Medic Lines

*Medicago truncatula* was transformed using the EHA105 *A. tumefaciens* strain harboring the CRISPR/Cas9 binary vectors targeting the *CYP93E2* or the *CYP72A61* genes. After several weeks on selective embryo inducing and proliferating media no somatic embryos were isolated from leaf explants co-cultivated with EHA105 carrying the *CYP72A61* targeting construct. Differently, somatic embryos were produced from embryogenic calli obtained from explants transformed with the *CYP93E2* targeting construct (Supplementary Figure 2; Supplementary Table 3). Fifty-two putative *CYP93E2* mutant plant lines were recovered with a transformation efficiency of 6.4%. Fourteen kanamycin-resistant plant lines transformed with the *Cas9* and *nptII* genes without the gRNAs (control) were also regenerated, with a comparable transformation efficiency (4.4%). The genome-edited and control plant lines were found to be phenotypically similar with no visible growth abnormalities during *in vitro* micropropagation and acclimation to soil.

### CRISPR/Cas9 Induces Highly Efficient Mutagenesis of the *CYP93E2* Gene

**Figure 2:**
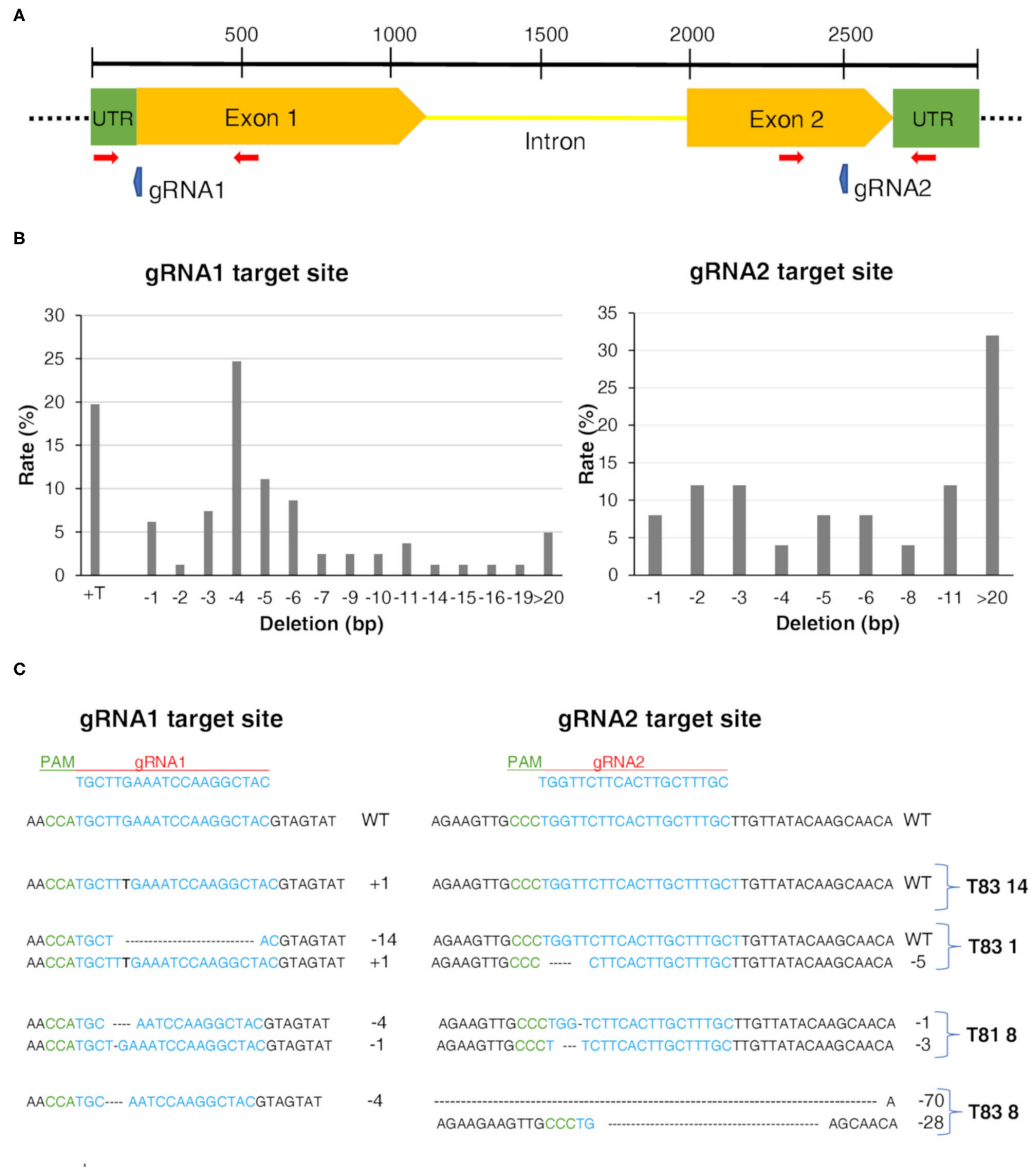
CRISPR/Cas9-editing in *Medicago truncatula*. **(A)** Gene structure of *CYP93E2* with target sites of CRISPR/Cas9 gRNAs (blue arrows) designed in the first and second exon. The red arrows represent primers used to screen mutations. **(B)** Rate of mutation sizes. Percentages were calculated by dividing the number of events of each mutation size by the sum of total mutation events. **(C)** Representation of mutation events generated by the CRISPR/Cas9 system in *CYP93E2* gene of the four selected plant lines. Mutations were detected using as reference the control plant lines. The *CYP93E2* target sequences are colored in blue. Within the sequence alignment, deletions are represented by traits; insertions are shown with black bold letters. Mutation size and plant line ID are shown on the right. Guide RNA (gRNA) and protospacer adjacent motif (PAM) are indicated in red and green, respectively.

To determine whether the target *CYP93E2* gene was mutated, we genotyped 51 independent putative *CYP93E2* mutants and three control T0 plant lines using specific primers for the *Cas9* gene and for regions flanking the *CYP93E2* target sequences (Supplementary Table 2; Figure 2A). All the 51 mutants and the three control plant lines showed the genomic integration of the transgene. Sequencing and chromatogram decomposition analysis showed that, compared with the wild type sequence, 43 out of the 51 mutant plant lines (84.3%) carried mutations in at least one of the two targeted sites of the *CYP93E2* gene (Supplementary Table 4). Considering gRNA1, all the 43 edited lines showed target sequence mutations. Among these, 21 lines were completely edited showing a homozygous mutation or biallelic profiles. The other 22 lines had a partially edited genotype (heterozygous) and retained a proportion of the wild type allele (Supplementary Table 4). At the gRNA2 target locus, a lower editing efficiency was observed as 25 of the 43 edited plant lines retained the wild type sequence. In 11 lines a partially edited genotype (heterozygous) was found and only seven lines were completely edited showing a homozygous mutation or biallelic profiles (Supplementary Table 4). The most frequent types of mutation at the gRNA1 target site were a −4 bp deletion and +T insertion (Figure 2B), while at the gRNA2 target site 32% of the observed mutations were long bp deletions. After identifying the targeted mutations, we randomly selected four (T81 8, T83 1, T83 8, and T83 14) T0 mutant lines based on the different type mutations at the target sites of the *CYP93E2* gene. Focusing on gRNA1, two were biallelic mutants and two homozygous. Focusing on gRNA2, two were biallelic mutants, one heterozygous and one wild type. The edited sequences of the selected plant lines are shown in Figure 2C. The deduced amino acid sequences of the CYP93E2 protein for the selected lines show frame-shifting with the introduction of amino acid mutations in the catalytic site and stop codons responsible for the premature termination of protein translation (T81 8, T83 8) or the loss of the ATG starting codon (T83 1, T83 14).

### Phenotypic and Morphological Observations of *CYP93E2* Mutant Barrel Medic Lines

**Figure 3:**
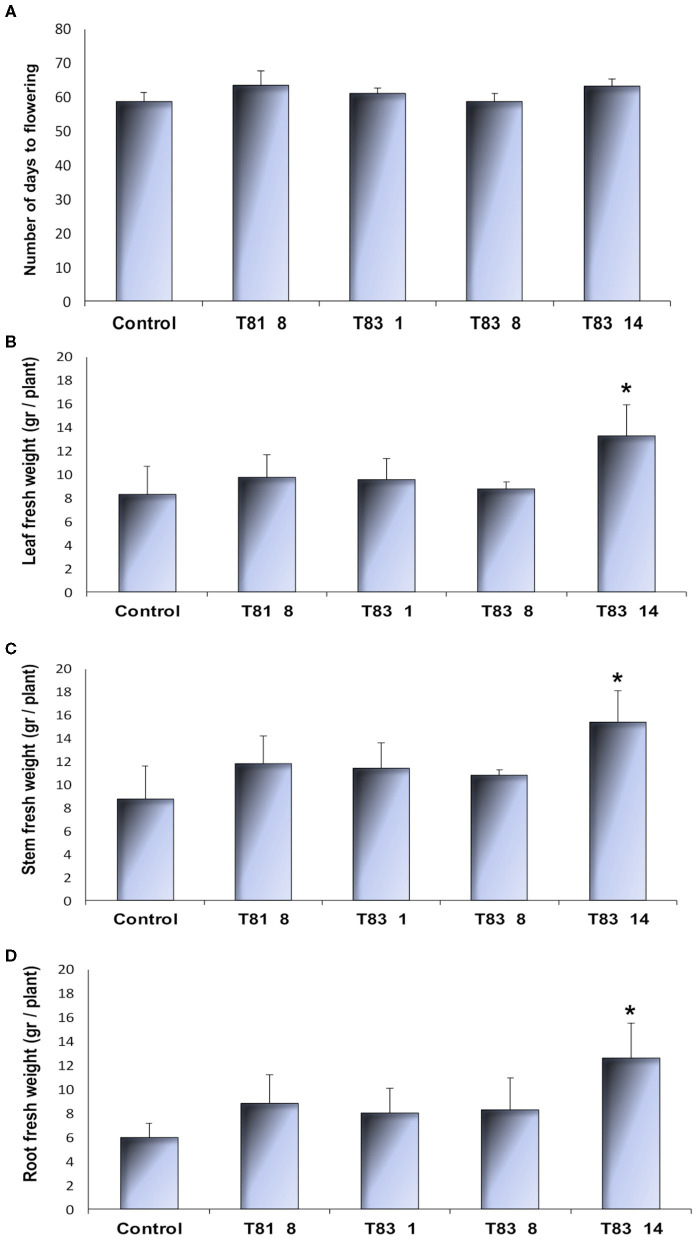
Morphological observations of control and selected *CYP93E2* mutant barrel medic plant lines under greenhouse conditions. **(A)** Number of days from transplanting to flowering. **(B–D)** Leaf, stem and root fresh weight per plant at the start of plant flowering, respectively. The greenhouse experiment was performed with four biological replications of single-plant experimental units. Data are shown as the mean values with error bars representing standard deviation. An asterisk indicates means significantly different from control according to Dunnett's Test (*p* < 0.05).

*CYP93E2* mutant and control plant lines showed similar morphological features, flowered and produced seeds under controlled environmental conditions. All tested plants showed root nodulation with no visible differences between the control and the *CYP93E2* mutants. To investigate whether mutations in the *CYP93E2* gene affect the plant growth and yield, we characterized the four selected T0 mutant lines by measuring the number of days from transplanting to flowering and leaf, stem and root biomass at the start of the flowering period. All the tested mutants showed a similar flowering time than the control (Figure 3A). Three mutant lines (T81 8, T83 1, and T83 8) showed plant growth performances similar to the control while the fourth mutant (T83 14) exhibited a significant increase in plant biomass production, according to Dunnett's test (Figures 3B–D; Supplementary Figure 3). The results obtained for the dry weight of shoot and root biomass confirmed the data of the fresh weight measurements (Supplementary Figure 4).

### Knock-Out of the *CYP93E2* Gene by CRISPR/Cas9 Affect Both Non-hemolytic and Hemolytic Sapogenin Contents

**Figure 4:**
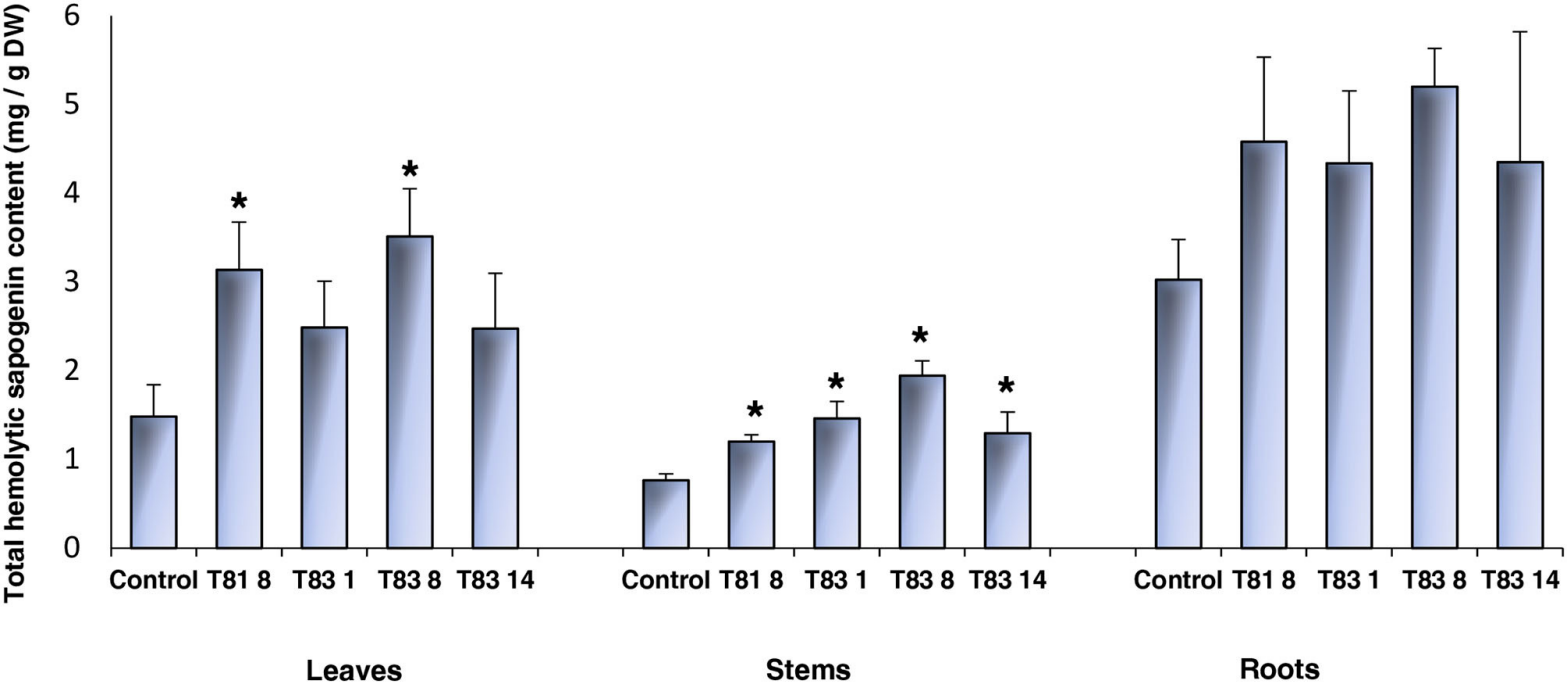
Total hemolytic sapogenin content in leaves, stem and roots of control and *CYP93E2* mutant barrel medic plants under greenhouse experimental conditions. Data are shown as the mean values of four biological replications of single-plant experimental units with error bars representing standard deviation. An asterisk indicates means significantly different from control according to Dunnett's Test (*p* < 0.05).

To investigate the effects of knock-out mutations in the *CYP93E2* gene on the triterpene saponin content of leaves, stems and roots, all selected mutant lines were biochemically analyzed. All sapogenins were identified by using authentic reference standards and quantitated considering all the obtained sapogenins (Tava et al., 2017) (Supplementary Figure 1). The results of this investigation are shown in Tables 1, 2 where the amount (mg/g DM) of each identified sapogenin is reported for aerial parts (leaves and stems) and roots, respectively. The hemolytic sapogenin composition of all *CYP93E2* mutant lines is similar to the control, while no soyasapogenols have been detected in any of the analyzed organs. The ANOVA revealed significant differences (*p* < 0.0001) among the barrel medic lines for the soyasapogenol B and A contents in the leaves, stems and roots (Tables 1, 2). According to Dunnett's test, all mutant lines showed significant differences in the content of both soyasapogenols compared to the control. Considering the total content of hemolytic sapogenins in leaves, the ANOVA revealed significant differences (*p* = 0.005) among the barrel medic lines. Two (T81 8 and T83 8) mutant lines showed a significantly higher total hemolytic sapogenin content, according to Dunnett's Test (Table 1; Figure 4). All mutant lines displayed significant higher amounts of medicagenic acid when compared to the control. Furthermore, three (T81 8, T83 1, and T83 8) mutant lines showed significantly higher bayogenin level, and two (T831 and T83 8) of them also displayed a significant increase of zanhic acid content than the control (Table 1). The amount of medicagenic acid and zanhic acid, the major hemolytic sapogenins in *M. truncatula* aerial parts, was more than two times higher in T81 8 and T83 8 mutants compared to the control. The total leaf sapogenin content varied in the range 2.47–3.51 mg/g DM in *CYP93E2* mutants and was not significantly different from the control line (3.13 mg/g DM). According to Dunnett's Test, all mutant lines displayed significant higher total amounts of hemolytic sapogenins in the stems when compared to the control (Table 1; Figure 4). As seen in the leaves, all tested lines showed significantly higher medicagenic acid level, and the same two lines (T81 8 and T83 8) displayed a significant increase of zanhic acid content than the control. With regard to total sapogenin content in the stems, T83 8 displayed a similar level to the control, while the other three mutant lines showed a significant reduction (Table 1). Differently from the aerial part, in the roots the hemolytic sapogenins oleanolic acid and 2β-hydroxy oleanolic acid were also detected in both the control and edited lines (Table 2). The content of all hemolytic sapogenins in roots, including oleanolic acid, 2β-hydroxy oleanolic acid, hederagenin, bayogenin and medicagenic acid, was higher in all mutants than in the control. Specifically, two (T81 8 and T83 8) mutant lines showed significantly higher hederagenin content, and one (T83 8) of them also displayed a significant increase of medicagenic acid content than the control, according to Dunnett's Test. No significant differences were observed between *CYP93E2* mutants and the control for the total sapogenin content in roots. Interestingly, the biochemical characterization revealed the presence of β-amyrin (0.08 mg/g DM) in the roots of all mutant lines but not in the control, and these differences were significant, according to Dunnett's test (Table 2).

**Table 1:** Sapogenin content in aerial parts (leaves and stems) of control and *CYP93E2* mutant barrel medic plant lines under greenhouse experimental conditions.

**Plant organ**	**Compound**	**Plant line**					**Statistics**		
		**Control**	**T81 8**	**T83 1**	**T83 8**	**T83 14**	***F***	**(df)**	**Probability**
Leaves	Bayogenin	0.015 ± 0.002	0.034 ± 0.005*	0.032 ± 0.005*	0.047 ± 0.009*	0.028 ± 0.007	10.31	4	0.0007
	Medicagenic acid	0.73 ± 0.14	1.58 ± 0.20*	1.45 ± 0.24*	1.85 ± 0.26*	1.28 ± 0.27*	10.06	4	0.0008
	Zanhic acid	0.74 ± 0.22	1.52 ± 0.35*	1.00 ± 0.27	1.62 ± 0.27*	1.17 ± 0.35	4.56	4	0.018
	Soyasapogenol A	0.07 ± 0.02	0.00*	0.00*	0.00*	0.00*	51.86	4	<0.0001
	Soyasapogenol B	1.57 ± 0.45	0.00*	0.00*	0.00*	0.00*	44.80	4	<0.0001
	Total hemolytic sapogenins	1.49 ± 0.36	3.14 ± 0.54*	2.49 ± 0.52	3.51 ± 0.54*	2.47 ± 0.62	6.53	4	0.005
	Total non-hemolytic sapogenins	1.64 ± 0.47	0.00*	0.00*	0.00*	0.00*	45.16	4	<0.0001
	Total sapogenins	3.13 ± 0.80	3.14 ± 0.54	2.49 ± 0.52	3.51 ± 0.54	2.47 ± 0.62	1.84	4	0.19
Stems	Medicagenic acid	0.70 ± 0.05	1.08 ± 0.08*	1.38 ± 0.17*	1.74 ± 0.16*	1.20 ± 0.21*	19.52	4	<0.0001
	Zanhic acid	0.06 ± 0.02	0.12 ± 0.03*	0.08 ± 0.02	0.20 ± 0.01*	0.10 ± 0.03	15.91	4	<0.0001
	Soyasapogenol A	0.08 ± 0.01	0.00*	0.00*	0.00*	0.00*	260.4	4	<0.0001
	Soyasapogenol B	1.33 ± 0.31	0.00*	0.00*	0.00*	0.00*	67.61	4	<0.0001
	Total hemolytic sapogenins	0.77 ± 0.07	1.20 ± 0.08*	1.46 ± 0.19*	1.95 ± 0.17*	1.30 ± 0.24*	20.30	4	<0.0001
	Total non-hemolytic sapogenins	1.41 ± 0.32	0.00*	0.00*	0.00*	0.00*	73.41	4	<0.0001
	Total sapogenins	2.17 ± 0.39	1.20 ± 0.08*	1.46 ± 0.19*	1.95 ± 0.17	1.30 ± 0.24*	11.57	4	0.0004

*For each barrel medic line, data represent the mean values (mg/g DM) ± SD of four replicated plants. An asterisk indicates means significantly different from control according to Dunnett's test (p < 0.05)*.

**Table 2:** Sapogenin content in roots of control and *CYP93E2* mutant barrel medic plant lines under greenhouse experimental conditions.

**Compound**	**Plant line**					**Statistics**		
	**Control**	**T81 8**	**T83 1**	**T83 8**	**T83 14**	***F***	**(df)**	**Probability**
β-amyrin	nd	0.08 ± 0.02*	0.08 ± 0.03*	0.08 ± 0.01*	0.08 ± 0.01*	15.12	4	0.0001
2β-hydroxy oleanolic acid	0.20 ± 0.06	0.21 ± 0.04	0.24 ± 0.04	0.26 ± 0.03	0.25 ± 0.13	0.45	4	0.77
Oleanolic acid	0.26 ± 0.05	0.35 ± 0.05	0.36 ± 0.06	0.40 ± 0.06	0.36 ± 0.13	1.37	4	0.30
Hederagenin	0.66 ± 0.06	1.20 ± 0.25*	0.99 ± 0.20	1.30 ± 0.06*	1.10 ± 0.29	4.26	4	0.02
Bayogenin	0.60 ± 0.15	0.81 ± 0.19	0.85 ± 0.15	0.94 ± 0.08	0.85 ± 0.42	0.78	4	0.56
Medicagenic acid	1.31 ± 0.15	2.02 ± 0.46	1.90 ± 0.42	2.30 ± 0.25*	1.79 ± 0.51	2.46	4	0.10
Soyasapogenol A	0.01 ± 0.003	0.00*	0.00*	0.00*	0.00*	36.82	4	<0.0001
Soyasapogenol B	1.37 ± 0.27	0.00*	0.00*	0.00*	0.00*	92.47	4	<0.0001
Total hemolytic sapogenins	3.03 ± 0.46	4.58 ± 0.95	4.34 ± 0.82	5.20 ± 0.43	4.35 ± 1.47	2.02	4	0.15
Total non-hemolytic sapogenins	1.38 ± 0.28	0.00*	0.00*	0.00*	0.00*	91.77	4	<0.0001
Total sapogenins	4.40 ± 0.39	4.58 ± 0.95	4.34 ± 0.82	5.20 ± 0.43	4.35 ± 1.47	0.44	4	0.78

*For each barrel medic line, data represent the mean values (mg/g DM) ± SD of four replicated plants. An asterisk indicates means significantly different from control according to Dunnett's test (p < 0.05)*.

### Editing of *CYP93E2* Affected Expression of Genes Involved in the Saponin Pathway

**Figure 5:**
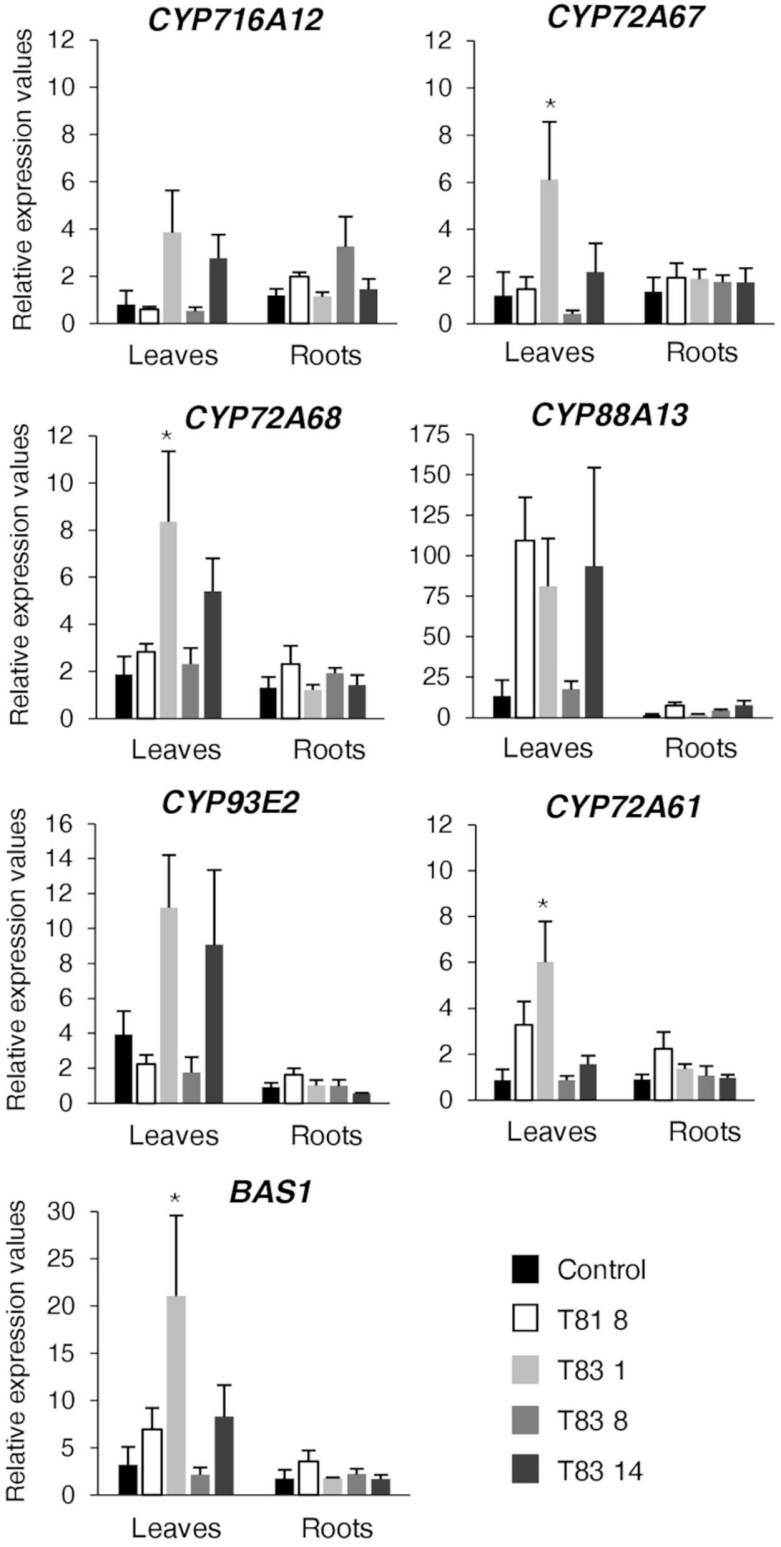
Expression analysis using quantitative RT-PCR on sapogenin pathway genes. Values were calculated by the comparative Ct (cycle threshold) method using roots of control line as reference. Values are means ± SE of four biological replicates. An asterisk indicates means significantly different from control according to Dunnett's Test (*p* < 0.05).

Quantitative real-time PCR analysis was performed on leaves and roots of the control and all the selected mutants for the β-amyrin synthase *BAS1* gene (Suzuki et al., 2002), for the genes of the hemolytic sapogenin branch - *CYP716A12* (Carelli et al., 2011), *CYP72A68* (Fukushima et al., 2013), *CYP72A67* (Biazzi et al., 2015), *CYP88A13* (Ribeiro et al., 2020) and for the genes of the non-hemolytic sapogenin branch - *CYP93E2* (Fukushima et al., 2011) and *CYP72A61* (Fukushima et al., 2013). The results are expressed as relative expression values using the roots of the control line as reference. As regards the genes of the hemolytic pathway, Dunnett's Test revealed a significantly enhanced accumulation of *CYP72A67* and *CYP72A68* transcripts in the leaves of T83 1 mutant (Figure 5). Although ANOVA revealed no significant differences (*p* = 0.08) in *CYP716A12* expression in the leaves of barrel medic lines, higher expression levels were detected for T83 1 and T83 14 mutants compared to the control. Interestingly, *CYP88A13*, the gene responsible for the synthesis of zanhic acid, was highly induced in leaves of three (T81 8, T83 1, and T83 14) mutant lines. Zanhic acid represent the last step of hemolytic saponin branch (Figure 1) and together with medicagenic acid were the most abundant sapogenins in leaves of *CYP93E2* mutant lines (Table 1). The up-regulation of genes involved in the hemolytic sapogenin biosynthesis in some of the edited barrel medic lines was in accordance with the increased levels of hemolytic sapogenins found. As regards the genes of the non-hemolytic branch, two (T831 and T83 14) mutant lines showed a higher amount of *CYP93E2* transcript in leaves, and one (T83 1) of them also displayed a significant *CYP72A61* up-regulation when compared to the control (Figure 5). Concerning the *BAS1* expression level in leaves, the ANOVA revealed significant differences (*p* = 0.04) among the tested lines and T83 1 showed a significant *BAS1* up-regulation when compared to the control. No significant differences in the expression profile of both hemolytic and non-hemolytic genes in roots were observed among barrel medic lines (Figure 5).

## Discussion

Metabolic engineering of triterpene saponin biosynthesis in plants is a fascinating research topic from two main perspectives. The wide potential use of saponins as biologically and pharmacologically active compounds, and their possible effects on fundamental plant developmental processes, make their modulation an interesting tool for breeding and biotechnology. Also, the development of plants with modified saponin profiles could yield further understanding of their biosynthetic route and regulation mechanisms. The metabolic engineering of both the early and late stages of the saponin biosynthetic pathway has been the topic of several reviews (Yendo et al., 2010; Lambert et al., 2011; da Silva Magedans et al., 2020). The major requirement for the successful engineering of specific saponins is the knowledge of the genes/enzymes involved in their biosynthesis. Over recent years, the economic relevance of these valuable natural compounds led to a substantial understanding of the biosynthetic routes of triterpene saponins, in particular in *M. truncatula*, a rich source of different saponins and a model plant species for legumes (Pateraki et al., 2015; Carelli et al., 2020). Several members of P450 families have been characterized to be involved in saponin biosynthesis of *M. truncatula* (Carelli et al., 2011; Fukushima et al., 2011, 2013; Biazzi et al., 2015; Ribeiro et al., 2020) but the *in planta* function of a limited number of these P450s was determined. Furthermore, results from studies with heterologous microbial systems do not always correspond with *in planta* function. By using a yeast expression system, Biazzi et al. (2015) supported the hypothesis that CYP72A68 is mainly involved in the biosynthesis of medicagenic acid, while Tzin et al. (2019) showed that CYP72A68 is a multi-functional oxidase responsible for hederagenin, gypsogenin and gypsogenic acid based on *in vivo* and *in planta* evidence. To date, the functional roles *in planta* of CYP93E2 and CYP72A61, which participate in the biosynthesis of non-hemolytic sapogenins in *M. truncatula*, have not been demonstrated yet. The biochemical functions of these P450 key enzymes and the physiological roles of saponins in barrel medic could be elucidated by characterization of knock-out mutants or gene over-expression. Few studies reported the CRISPR/Cas9-mediated genome editing in *Medicago* spp. (Michno et al., 2015; Curtin et al., 2017, 2018; Meng et al., 2017; Gao et al., 2018; Wang et al., 2018; Yu et al., 2018; Wolabu et al., 2020) and none of them involved specific genes associated with the metabolism of saponins.

In this work, we used the CRISPR/Cas9 technology in the model legume *Medicago truncatula* to knock-out two key CYP450 genes of the non-hemolytic saponin pathway (*CYP93E2* and *CYP72A61*), in order to demonstrate their specific role *in planta* and explore their possible involvement in growth and development. We adopted a dual gRNA approach for targeting *CYP93E2* and *CYP72A61* in order to enhance the genome editing efficiency by either increasing the probability of successful mutagenesis, or even deleting large gene fragments (Nian et al., 2017; Pauwels et al., 2018; Do et al., 2019), and we made use of assembly of the GoldenBraid GB cloning system suited for gene editing experiments (Vazquez-Vilar et al., 2016; Aliaga-Franco et al., 2019; Maioli et al., 2020). In this study, we demonstrate for the first time that *CYP93E2* can be efficiently edited in *M. truncatula*, resulting in the manipulation of the saponin metabolic pathway. Heterozygous, biallelic and homozygous mutants were detected in T0 plants, suggesting that editing occurred at an early stage of the transformation/regeneration processes (Li et al., 2018). The overall gene editing efficiency was 84%, higher than what observed in other published works in *M. truncatula* (Meng et al., 2017; Curtin et al., 2018; Wolabu et al., 2020). All the 43 edited lines showed mutations at the gRNA1 locus. The gRNA2 locus showed a lower mutation rate, with 25 of the 43 (58%) edited plant lines not showing any editing effect (Supplementary Table 4). Although the editing efficiency was different between the two gRNAs, it was not possible to attribute this discrepancy to the different GC contents of the target sequences or sgRNA secondary structure or promoters that direct Cas9 and gRNAs expression (Ma et al., 2015). Interestingly, the most frequent types of mutation at the *CYP93E2* gRNA1 target site were a −4 bp deletion and the insertion of a single nucleotide, while at the gRNA2 target site 32% of the observed mutations were long bp deletions (Figure 2B). In *M. truncatula* deletion of 1–10 nucleotides were the most common mutations (Michno et al., 2015; Meng et al., 2017; Curtin et al., 2018). The mutation pattern is consistent with that predicted with CRISPOR (http://crispor.tefor.net/) based on microhomology. Off-target mutations have not proven to be a cause for concern in plants (Hahn and Nekrasov, 2019) contrary to what was observed in human cells (Fu et al., 2013). The presence of mismatches in the seed region between the selected sgRNA and the off-targets supports the mutation efficiency (Hahn and Nekrasov, 2019), since this 3' terminal region of the target sequence strongly affects recognition by Cas9. The prospective off-target activity with the gRNAs used in this work was predicted using CasOFFinder, and no loci were found in the *M. truncatula* genome which could be considered as a likely source of off-target gene editing (Supplementary Data 1). In the only 2 prospective off-target loci with fewer than 3 mismatches with respect to the gRNA sequence, these were located in the seed region, and would therefore highly impair recognition.

Among all *CYP93E2* T0 mutant plant lines, four representative lines were chosen for further phenotype observation and biochemical analyses. The results of greenhouse trial showed no significant differences between most of the tested mutant lines (T81 8, T83 1 and T83 8) and the control in shoot and root biomass (Figure 3). These plant lines exhibited normal flowering time and produced seeds. These results were similar to a previous report which showed that homozygous mutant lines of *Lotus japonicus* harboring *Lotus retrotrasposon1* (*LORE1*) in *LjCYP93E1*, an orthologous of the *M. truncatula CYP93E2*, did not accumulate soyasapogenols without significant effects on plant growth and reproduction (Suzuki et al., 2019). The same authors suggest that the soyasaponins are not necessary for plant growth in *Lotus japonicus*. Biazzi et al. (2015) reported that TILLING barrel medic mutants of CYP72A67, a key enzyme in the biosynthesis of hemolytic sapogenins in *M. truncatula*, accumulated gypsogenin, gypsogenic acid, and 16α-hydroxy gypsogenic acid instead of medicagenic and zanhic acids in the leaves, without affecting plant growth and reproduction. Krishnamurthy et al. (2019) reported the modification of saponin composition in soybean mutants isolated from a high-density soybean mutant library targeted to β-amyrin synthase, and showed that mutant plants grow normally. Furthermore, Confalonieri et al. (2009) showed that the ectopic expression of a β-amyrin synthase-encoding sequence (*AsOXA1*) in transgenic *M. truncatula* led to higher accumulation of triterpene sapogenins and enhanced root nodulation without significant effects on plant growth and biomass. Unexpectedly, a fourth *CYP93E2* mutant line (T83 14) showed a significant enhancement of biomass production, and exhibited flowering time and seed recovery similar to the control and the other *CYP93E2* mutants (Figure 3). These specific results differ from previous reports which revealed detrimental effects on plant growth of *M. truncatula* associated to a mutation of genes encoding P450s or UDP- glycosyltransferases involved in hemolytic saponin biosynthesis (Naoumkina et al., 2010; Carelli et al., 2011). The differences in plant growth performances observed for T83 14 mutant line could result from positional effects or somaclonal variation which are not unusual in the production of transgenic plants and therefore cannot be excluded. In conclusion, no adverse influence was observed on the plant morphological features of *CYP93E2* mutants under greenhouse conditions.

To examine in detail the metabolic changes between the control and *CYP93E2* mutant barrel medic lines, we compared the triterpenoid sapogenin composition of the leaves, stems and roots. Our objective was to generate *CYP93E2 M. truncatula* knock-out mutants in which the metabolic flux to non-hemolytic saponins is totally blocked, with a potential impact on valuable hemolytic saponin levels. Gene editing tools allowed us to obtain mutants with a homogeneous genetic background, without stacking other mutations. As expected, all tested *CYP93E2* mutants did not accumulate soyasapogenols in any aerial and subterranean organs. These results provide the first *in planta* demonstration of the key role of *CYP93E2* in the control of triterpenoid saponin accumulation, confirming the results of yeast heterologous expression studies by Fukushima et al. (2011) and Moses et al. (2014b), and are consistent with previous findings reported for other legume species (Suzuki et al., 2019). So, in *Medicago truncatula CYP93E2* is directly involved in soyasaponin biosynthesis by catalyzing the C-24 hydroxylation of the β-amyrin backbone and its loss of function is sufficient to completely suppress non-hemolytic sapogenin biosynthesis. Sophoradiol, a β-amyrin derivative oxidized at the C-22 position by the CYP72A61, was not detected in aerial and subterranean organs of all *CYP93E2* mutant and control lines. Differently, Suzuki et al. (2019) showed accumulation of sophoradiol in *LyCYP93E1 Lotus japonicus* mutants, and Krishnamurthy et al. (2019) detected this compound in *CYP93E1* soybean mutants. In particular, Suzuki et al. (2019) proposed for *L. japonicus* a triterpenoid biosynthetic pathway with two possible paths starting from β-amyrin toward soyasapogenol B, one of which is catalyzed by LjCYP93E1. Our results provide the evidence that CYP93E2 is the first and only enzyme responsible for the first key oxidative step of non-hemolytic saponin pathway in *M. truncatula*. The differences between results from *L. japonicus* and soybean and the ones observed in our study might be related to the physiological differences between the legume species and to the different plant organs (leaves, stems and roots vs. hypocotyls and cotyledons).

Knocking-out *CYP93E2* influenced the biosynthetic pathway of hemolytic saponins and resulted in the redirection of metabolic fluxes in all the tested mutant lines. In fact, the leaves of all *CYP93E2* mutant lines accumulated higher amounts of total hemolytic sapogenins than those of the control plants (Figure 4). In T81 8 and T83 8 mutants, the plant lines with the significantly higher total hemolytic sapogenin content, the amount of medicagenic and zanhic acid, known as the major hemolytic sapogenins in *M. truncatula* aerial parts (Kapusta et al., 2005; Tava and Avato, 2006; Lei et al., 2019), was more than doubled with respect to the control. Furthermore, the total leaf sapogenin content of all *CYP93E2* mutants was not significantly different from the control line. In stems, despite the significant increase in hemolytic sapogenins in all the tested mutants, most of them did not totally compensate for the absence of soyasapogenols. In roots, the content of hemolytic sapogenins was less variable with only two mutants (T81 8 and T83 8) showing significantly higher hederagenin content than the control, and T83 8 having a significantly higher medicagenic acid content (Table 2). Overall, these results indicate that the absence of soyasapogenols induced a compensation by the increased accumulation of hemolytic sapogenins (Figure 4). Suzuki et al. (2019) showed an increase of oleanolic acid only in the roots of *LyCYP93E1* mutant plants, while our quantitative biochemical data clearly indicate that the absence of soyasapogenols in the *MtCYP93E2* mutants induced an increase in the accumulation of hemolytic sapogenins in all tested organs. The plant response to the absence of hemolytic sapogenins was not the same; in fact, Carelli et al. (2011) demonstrated that knock-out mutants of CYP716A12, a key enzyme catalyzing the first step of hemolytic sapogenin pathway, blocked the synthesis of all hemolytic sapogenins without significant and consistent effects on soyasapogenol levels. Interestingly, the biochemical characterization revealed the presence of β-amyrin in the roots of all mutant plant lines, but not in the control. The significant accumulation of β-amyrin, a common precursor of both hemolytic and non-hemolytic sapogenins, could be the direct consequence of the metabolic block toward soyasapogenol production imposed on *CYP93E2* mutants, and this result is consistent with previous findings (Suzuki et al., 2019). Differently, β-amyrin was not detected in the leaves and stems of *CYP93E2* mutants, suggesting that this precursor is entirely converted to hemolytic sapogenins.

To verify whether *CYP93E2* mutations had caused changes in gene expression, qRT-PCR was used to detect the expression levels of genes related to hemolytic and non-hemolytic sapogenin pathway. All the tested mutant lines partially or totally compensate the absence of soyasapogenols by increasing the content of hemolytic sapogenins in order to maintain a total sapogenin “threshold” content similar to the control (Tables 1, 2; Figure 4). Overall, the increased content of hemolytic sapogenins was not directly correlated with the expression of the corresponding genes, except for the T 83 1 and T83 14 mutants. The reason of this discrepancy could be that at the sampling time of analysis (start of flowering) the edited lines had already reached the total sapogenin “threshold,” so the corresponding genes did not appear induced with respect to the control line. The hypothesis of a “threshold” value for sapogenin content is reinforced considering the role of *CYP88A13*, the gene responsible for the conversion of medicagenic to zanhic acid, highly induced in leaves of the *CYP93E2* mutant lines. The conversion of medicagenic to zanhic acid could act as a leaf-specific way to maintain medicagenic acid within a genetically determined range. In fact, zanhic acid saponins showed lower hemolytic activities compared with medicagenic acid saponins (Oleszek et al., 1992), resulting in a reduced ability to cause membrane perturbation. The mutant lines showed higher levels of hemolytic sapogenins and in particular of medicagenic acid compared to the control line so the induction of *CYP88A13* can be part of this balance mechanism. Evidence of this mechanism was found in leaves of *Medicago* inter-specific hybrid derivatives (Carelli et al., 2015), where no correlation between zanhic acid and the other hemolytic sapogenin content was found, but a strict control of medicagenic/zanhic ratio was evidenced. Differently from leaves and stems, roots were characterized by a larger number of aglycones; among these, the precursors of medicagenic acid (Figure 1; Tables 1, 2) in the control line represented 57% of the total hemolytic sapogenins with respect to 1% in leaves. The edited lines compensated the absence of root soyasapogenols by increasing, though not significantly, all the classes of hemolytic sapogenins by the intervention of CYP716A12 (oleanolic acid), CYP716A12 and CYP72A67 (2β-hydroxy oleanolic acid), CYP716A12 and CYP72A68-mediated hydroxylation (hederagenin), CYP716A12, CYP72A67 and CYP72A68-mediated carboxylation (medicagenic acid) (Figure 1). Then, the absence of a prevailing hemolytic sapogenin biosynthetic target in root, as it is the case for medicagenic acid in leaves, lead to a more diversified expression pattern of the sapogenin biosynthetic genes in the edited lines that could account for the lack of significant differences from the control.

With regard to CRISPR/Cas9 mediated gene editing of *CYP72A61*, it was not possible to recover somatic embryos and regenerate transformed plantlets carrying mutations in the target sequences. In fact, when leaf explants with calli were transferred to a selective hormone-free medium, somatic embryos started to develop, but only in the control, pointing to an effect dependent on the role of the targeted gene. P450s are involved in the synthesis of secondary metabolites and are known for their roles in many important cell processes. In particular, they are involved in the regulation of plant hormone metabolism and they function directly in plant growth and development (Xu et al., 2015). The manipulation of the biosynthesis of saponins and their intermediates which accumulate during normal growth and development can determine morphological and physiological effects at various developmental stages, including seed germination, vegetative growth, flowering and embryo development, fruiting and nodulation, suggesting additional roles for these molecules in primary plant processes (Moses et al., 2014a). As far as we know, the only manuscript describing the effects of alteration of endogenous levels of triterpenes on embryogenesis was published by Go et al. (2012). In particular, knock-out *Arabidopsis* mutants for marneral synthase 1 (*MRN1*), which converts 2,3-oxidosqualene to the triterpene marneral, displayed altered levels of triterpenoids and sterols, delayed zygotic embryogenesis, reduced growth and late flowering (Go et al., 2012). It is possible that the mutation in *CYP72A61* is responsible for the absence of somatic embryo differentiation under selective *in vitro* conditions, presumably resulting from the altered flux balances among hormone and/or saponin biosynthesis, suggesting that *CYP72A61* might be involved in inhibition of embryo and shoot development and therefore important for plant growth and survival. Also, mutations of genes encoding P450s involved in saponin biosynthesis have revealed negative effects on plant growth processes that may be related to the accumulation of toxic intermediated (Moses et al., 2014a). CYP72A61 in M. truncatula catalyzes the hydroxylation of C-22 in 24-hydroxy-β-amyrin (the product of CYP93E2) to yield soyasapogenol B (Fukushima et al., 2013). A loss of function mutation in *CYP72A61* could presumably determine accumulation of 24-hydroxy-β-amyrin. This metabolic intermediate of soyasapogenols does not normally accumulate in the plant, and could result in toxic effects on somatic embryogenesis. To address both hypotheses, comprehensive molecular and metabolomic studies will be required to confirm the CYP72A61 mutations by CRISPR/Cas9 system and their effects at the cellular level, and the presence of 24-hydroxy-β-amyrin in undifferentiated calli.

In conclusion, we have assessed the *in planta* role of CYP93E2 in triterpene saponin biosynthesis in *M. truncatula*. The present study reports generation of *CYP93E2* barrel medic mutants with high efficiency by CRISPR/Cas9-mediated mutagenesis, and demonstrates for the first time the success of this strategy to block the metabolic flux toward non-hemolytic saponins, highlighting the potential for converting β-amyrin to valuable hemolytic saponins. These mutant plants with high content of hemolytic saponins are consistent with the potential industrial extraction of saponins for a wide range of applications. Our findings provide new and interesting insights into the application of CRISPR/Cas9 for metabolic engineering of high-value compounds of plant origin, offer the opportunity to develop new biological sources and additional useful informations to further investigate the physiological functions of saponins *in planta*.

## Data Availability Statement

The original contributions presented in the study are included in the article/Supplementary Material, further inquiries can be directed to the corresponding author/s.

## Author Contributions

MCo: conceived and planned the study, designed and performed genetic transformation, tissue culture and growth chamber/greenhouse experiments, analyzed the data, and wrote the manuscript. SG and AM: designed and cloned gene editing constructs and contributed to the revision of the manuscript. MCa: conducted PCR and QRT-PCR experiments, analyzed the data, and contributed to the revision of the manuscript. EB and AT: performed biochemical analyses. All authors contributed to the article and approved the submitted version.

## Conflict of Interest

The authors declare that the research was conducted in the absence of any commercial or financial relationships that could be construed as a potential conflict of interest.

## Publisher's Note

All claims expressed in this article are solely those of the authors and do not necessarily represent those of their affiliated organizations, or those of the publisher, the editors and the reviewers. Any product that may be evaluated in this article, or claim that may be made by its manufacturer, is not guaranteed or endorsed by the publisher.
