# Supplementary material for: CRISPR/Cas9-Mediated Targeted Mutagenesis of CYP93E2 Modulates the Triterpene Saponin Biosynthesis in Medicago truncatula
Source: Front Plant Sci. 2021 Jul 26;12:690231. doi: 10.3389/fpls.2021.690231 (PMC8350446; doi:10.3389/fpls.2021.690231)
Supplement: Supplementary file 1 [file Data_Sheet_1.PDF]

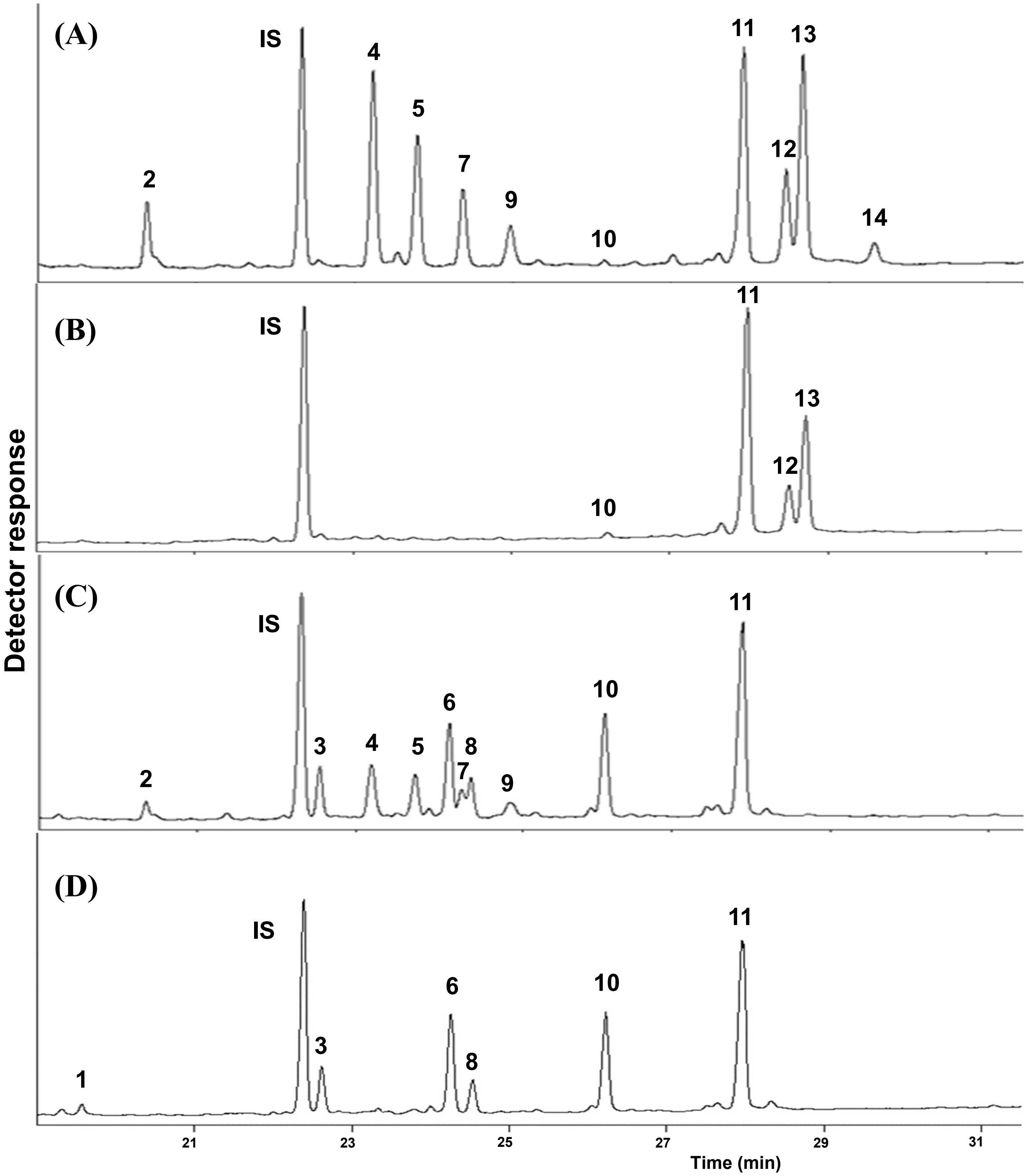

Supplementary Figure 1|Gas chromatogram of *M. truncatula* sapogenins. **(A)** control leaves. **(B)** T83 8 mutant leaves. **(C)** control roots. **(D)** T83 8 mutant roots. **IS** internal standard (uvaol). **1** *β*-amyrin. **2** soyasapogenol C. **3** oleanolic acid. **4** soyasapogenol D. **5** soyasapogenol F. **6** hederagenin. **7** 3*β*,22*β*,24-trihydroxyolean-18(19)-en. **8** 2*β*-hydroxyoleanolic acid. **9** soyasapogenol B. **10** bayogenin. **11** medicagenic acid. **12** zanhic acid. **13** 2*β*,3*β*,16*α*-trihydroxyolean-13(18)-en-23,28-dioic acid. **14** soyasapogenol A. For compound structure elucidation see Tava et al. (2017).
