# Supplementary material for: CRISPR/Cas9-Mediated Targeted Mutagenesis of CYP93E2 Modulates the Triterpene Saponin Biosynthesis in Medicago truncatula
Source: Front Plant Sci. 2021 Jul 26;12:690231. doi: 10.3389/fpls.2021.690231 (PMC8350446; doi:10.3389/fpls.2021.690231)
Supplement: Supplementary file 2 [file Data_Sheet_2.PDF]

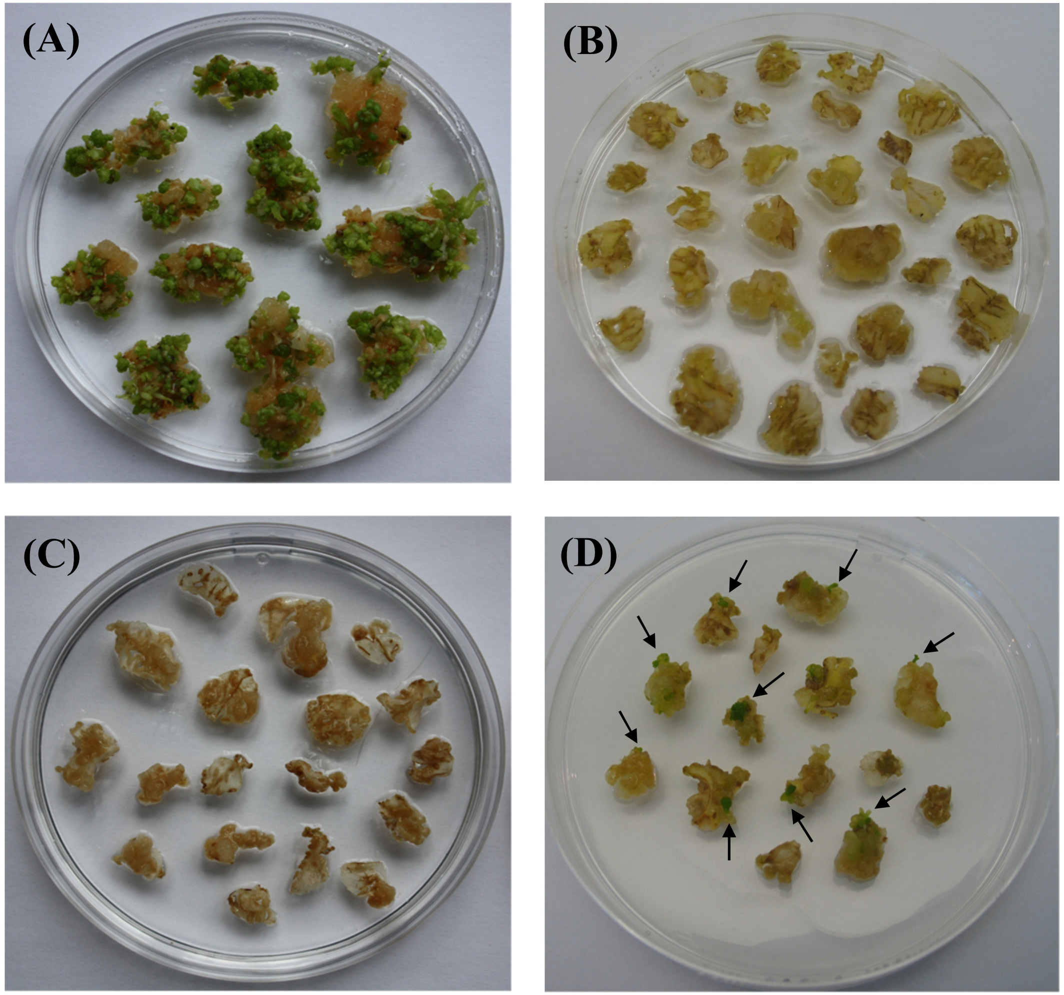


**Supplementary Figure 2 │** Genetic transformation and *in vitro* somatic embryogenesis of *Medicago truncatula* (M9-10a genotype). **(A)** Somatic embryogenesis from M9-10a leaf explants cultured on embryo induction medium (EIM) lacking kanamycin (positive control). **(B)** Leaf explants of M9-10a cultured on EIM containing kanamycin (negative control). **(C)** Leaf explants of M9-10a cultured on EIM with kanamycin after co-cultivation with EHA105 pCas9-CYP72A61 **(D)** Leaf explants of M9-10a cultured on EIM containing kanamycin after co-cultivation with EHA105 pCas9-CYP93E2. Black arrows indicate differentiation of somatic embryos on EIM selective medium.
