# Supplementary material for: CRISPR/Cas9-Mediated Targeted Mutagenesis of CYP93E2 Modulates the Triterpene Saponin Biosynthesis in Medicago truncatula
Source: Front Plant Sci. 2021 Jul 26;12:690231. doi: 10.3389/fpls.2021.690231 (PMC8350446; doi:10.3389/fpls.2021.690231)
Supplement: Supplementary file 3 [file Data_Sheet_3.PDF]

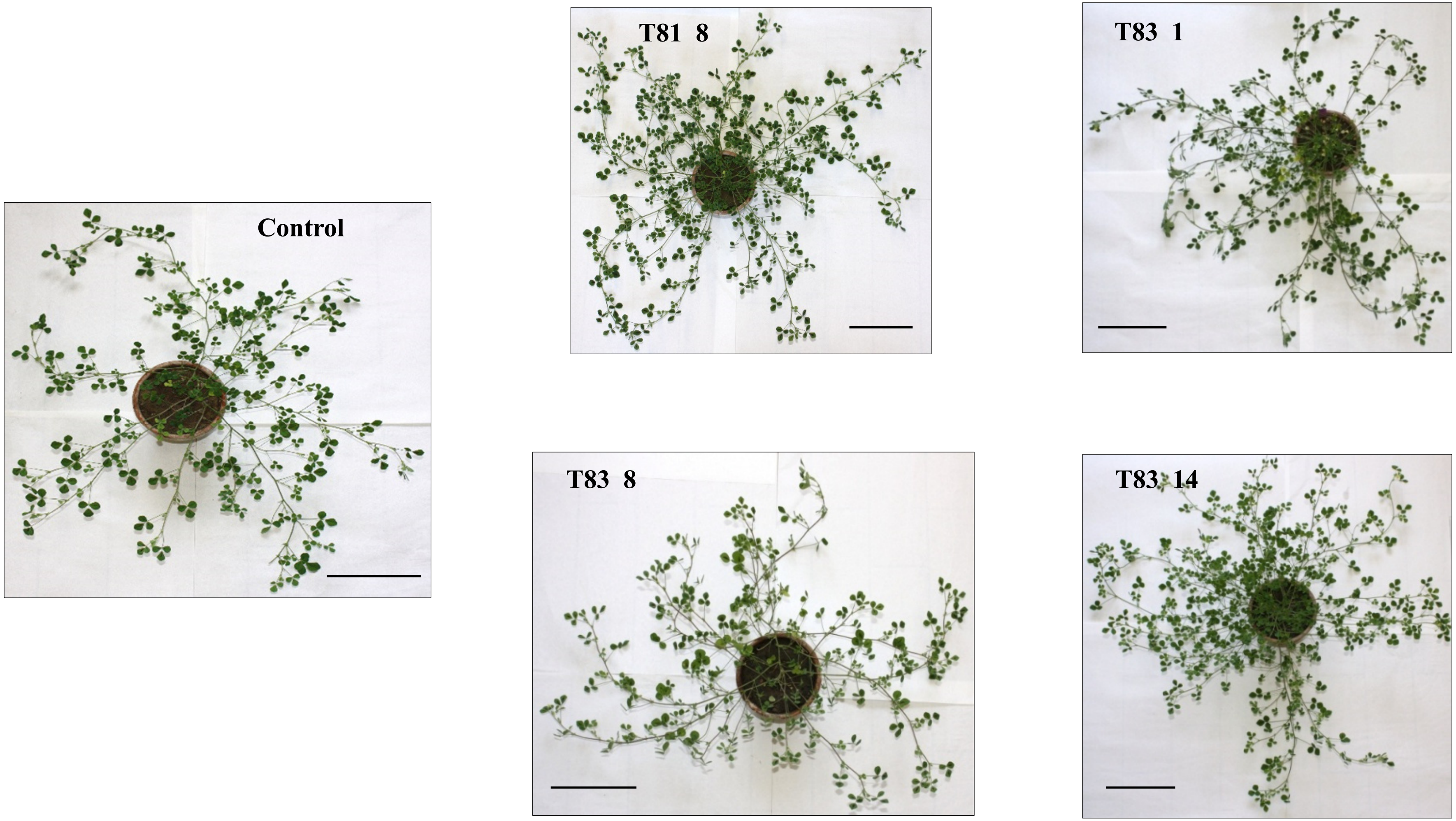

Supplementary Figure 3 | The phenotype of control and *CYP93E2* mutant (T81 8, T83 1, T83 8 and T83 14, respectively) plant lines under greenhouse conditions. Pictures were taken at the start of plant flowering. Scale bar = 12 cm.
