# Supplementary material for: CRISPR/Cas9-Mediated Targeted Mutagenesis of CYP93E2 Modulates the Triterpene Saponin Biosynthesis in Medicago truncatula
Source: Front Plant Sci. 2021 Jul 26;12:690231. doi: 10.3389/fpls.2021.690231 (PMC8350446; doi:10.3389/fpls.2021.690231)
Supplement: Supplementary file 4 [file Data_Sheet_4.PDF]

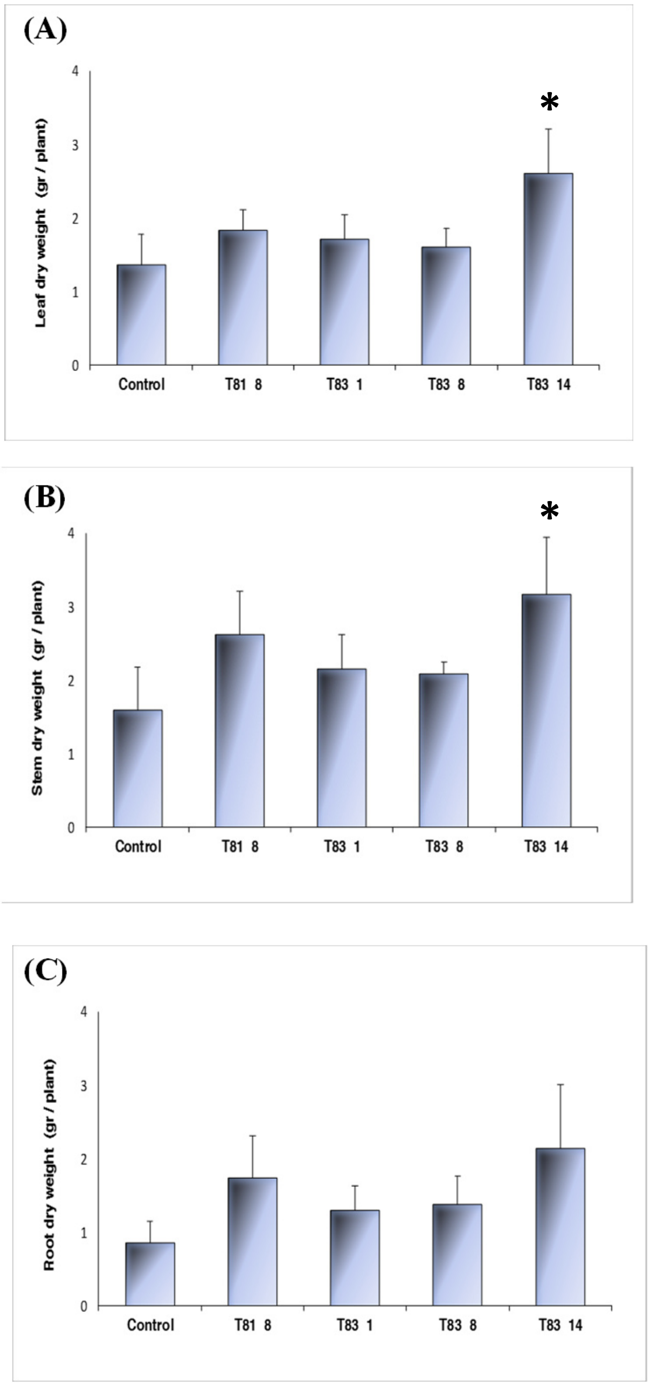

Supplementary Figure 4│Morphological observations of control and selected *CYP93E2* mutant barrel medic plant lines under greenhouse conditions. (A), (B) and (C) Leaf, stem and root dry weight per plant at the start of plant flowering, respectively. The greenhouse experiment was performed with four biological replications of single-plant experimental units. Data are shown as the mean values with error bars representing standard deviation. An asterisk indicates means significantly different from control according to Dunnett's test ($p < 0.05$).
