# Supplementary material for: CRISPR/Cas9-Mediated Targeted Mutagenesis of CYP93E2 Modulates the Triterpene Saponin Biosynthesis in Medicago truncatula
Source: Front Plant Sci. 2021 Jul 26;12:690231. doi: 10.3389/fpls.2021.690231 (PMC8350446; doi:10.3389/fpls.2021.690231)
Supplement: Supplementary file 5 [file Data_Sheet_5.PDF]

Supplementary Table 1|GoldenBraid parts and vectors used in this study. Their sequences and associated informations are available at https://gbcloning.upv.es/.

GB part	Description
GB1001	pUPD*At*U6-26 promoter
GB0645	pUPDpsgRNA (scaffold)
GB0639	pDGB2 alpha2 P35S:*h*Cas9:Tnos
GB1181	pDGB3 omega1 Tnos:nptII:Pnos
GB1657	pDGB3 omega1 Tnos:nptII:Pnos – P35S:*h*Cas9:Tnos
pDGB3 alpha1	pCambia Golden Braid destination vector
pDGB3 alpha2	pCambia Golden Braid destination vector
pDGB3 omega1	pCambia Golden Braid destination vector
pDGB3 omega2	pCambia Golden Braid destination vector
