# Supplementary material for: CRISPR/Cas9-Mediated Targeted Mutagenesis of CYP93E2 Modulates the Triterpene Saponin Biosynthesis in Medicago truncatula
Source: Front Plant Sci. 2021 Jul 26;12:690231. doi: 10.3389/fpls.2021.690231 (PMC8350446; doi:10.3389/fpls.2021.690231)
Supplement: Supplementary file 6 [file Data_Sheet_6.PDF]

Supplementary Table 2 | List of PCR primers.

Gene	Forward primer	Reverse primer
	qReal-time PCR	
Bas1	taccaattgcgctgctattcg	gcacgatggagaggagtagg
CYP716A12	ggatggaaaggacatcctga	ccaccatgcagtcacaagtt
CYP72A67	tggcagggcaagaatctact	taacaccaaatgctggtgga
CYP72A68	atgttgcagggcaagagact	ccggttacaggagggtacaa
CYP88A13	tggtaacatgcctaccttcgcc	catagctgaagcagggtatcctgg
CYP93E2	tttcatcaatgcatgggcta	tgaagaaccagggcaacttc
CYP72A61	catcccaggttacaggttcc	ctcattcctccaccaccatt
MSC27	cacccaaactagatgcagagaa	caccatccttgtagtaggcaaa
Actin	agtttgctattcaggccg	atttctcgctctgctgaggt
	Screening of edited plant lines	
Cas9	gagaaacaggagaaatcgtg	catgcttaacgtaattcaacag
1sgRNA	acttcactcaacagtaccattca	tcccactaagaagctcagtca
2sgRNA	actgttggcaaagaaaggct	tcctgccaagaagttttcaca
