# Supplementary material for: CRISPR/Cas9-Mediated Targeted Mutagenesis of CYP93E2 Modulates the Triterpene Saponin Biosynthesis in Medicago truncatula
Source: Front Plant Sci. 2021 Jul 26;12:690231. doi: 10.3389/fpls.2021.690231 (PMC8350446; doi:10.3389/fpls.2021.690231)
Supplement: Supplementary file 7 [file Data_Sheet_7.PDF]

Supplementary Table 3 | Transformation efficiency of M9-10a leaf explants co-cultivated with different *A. tumefaciens* strains: EHA105 p*nptII*-Cas9 (control), EHA105 pCas9-CYP93E2 and EHA105 pCYP72A61.

Bacterial Strain	No. of explants infected	No. of explants producing kanamycin-resistant somatic embryos	Transformation efficiency (%) *	No. of explants producing kanamycin-resistant plant lines	Transformation efficiency (%) **	No. of kanamycin-resistant plant lines tested by PCR (Cas9)	No. of kanamycin-resistant plant lines Cas9 +
EHA105 p*nptII*-Cas9	272	31	11.4	12	4.4	3	3
EHA105 pCas9-CYP93E2	529	54	10.2	34	6.4	52	51
EHA105 pCas9-CYP72A61	487	0	0	0	0	0	0

* The transformation efficiency was calculated dividing the number of leaf explants with kanamycin-resistant somatic embryos by the number of infected explants.

** The transformation efficiency was calculated dividing the number of leaf explants with kanamycin-resistant plant lines by the number of infected explants.
