# Supplementary material for: CRISPR/Cas9-Mediated Targeted Mutagenesis of CYP93E2 Modulates the Triterpene Saponin Biosynthesis in Medicago truncatula
Source: Front Plant Sci. 2021 Jul 26;12:690231. doi: 10.3389/fpls.2021.690231 (PMC8350446; doi:10.3389/fpls.2021.690231)
Supplement: Supplementary file 8 [file Data_Sheet_8.PDF]

**Supplementary Table 4 | ***CYP93E2* mutant T0 barrel medic plant lines: sequences at Sg1 and Sg2 target site.

Number of plant lines	Sg1 target site	Sg2 target site	Selected plant line
8	WT	WT	–
2	Heterozygous: WT and one long bp deletions	WT	–
6	Heterozygous: WT and one short bp deletions	WT	–
10	Heterozygous: WT and multiple mutated alleles	WT	–
4	Biallelic: short bp deletions	WT	–
1	Biallelic: -4bp deletion and +1bp insertion	WT	–
1	Homozygous: +1 bp insertion	WT	T83 14
1	Homozygous: -7 bp deletion	WT	–
2	Heterozygous: WT and one short bp deletions	Heterozygous: WT and short bp deletions	–
1	Heterozygous: WT and multiple mutated alleles	Heterozygous: WT and -55 bp deletions	_
3	Biallelic: short bp deletions	Heterozygous for long bp deletion	_
1	Biallelic: -4 bp deletion and -30 bp deletions	Heterozygous: WT and -1 bp deletion	_
2	Biallelic: short bp deletions	Heterozygous: WT and short deletion	_
2	Biallelic: short bp deletion and +1bp insertion	Heterozygous: WT and short bp deletion	T83 1
1	Heterozygous: WT and a long bp deletion	Biallelic: -4bp and -6bp deletions	_
1	Biallelic: -1bp and -4bp deletions	Biallelic: -1bp and -3bp deletions	T81 8
2	Homozygous: +1bp insertion	Biallelic: short bp deletions	–
1	Homozygous: -4bp deletion	Biallelic: -28bp and -70bp deletions	T83 8
1	Biallelic: -5bp deletion and +1bp insertion	Homozygous: -11bp deletion	_
1	Biallelic: -3bp and -4bp deletions	Homozygous: -126bp deletion	–
